# Supplementary material for: Weak Ultraviolet B Enhances the Mislocalization of Claudin-1 Mediated by Nitric Oxide and Peroxynitrite Production in Human Keratinocyte-Derived HaCaT Cells
Source: Int J Mol Sci. 2020 Sep 27;21(19):7138. doi: 10.3390/ijms21197138 (PMC7583049; doi:10.3390/ijms21197138)
Supplement: Supplementary file 1 [file ijms-21-07138-s001.pdf]

Supplementary Information

Weak ultraviolet B enhances the mislocalization of claudin-1 mediated by nitric oxide and peroxynitrite production in human keratinocyte-derived HaCaT cells

Mao Kobayashi [1]; Shokoku Shu [1], Kana Marunaka [1]; Toshiyuki Matsunaga [2], and Akira Ikari [1, *]

[1] Laboratory of Biochemistry, Department of Biopharmaceutical Sciences, Gifu Pharmaceutical University, Gifu 501-1196, Japan

[2] Education Center of Green Pharmaceutical Sciences, Gifu Pharmaceutical University, Gifu 502-8585, Japan

Running title: Mislocalization of claudin-1 by weak ultraviolet B in HaCaT cells

To whom correspondence should be addressed: Akira Ikari, Ph.D.
Laboratory of Biochemistry, Department of Biopharmaceutical Sciences,
Gifu Pharmaceutical University, 1-25-4 Daigaku-nishi, Gifu 501-1196, Japan,
Tel: +81-58-230-8124; Fax: +81-58-230-8124; E-mail: ikari@gifu-pu.ac.jp

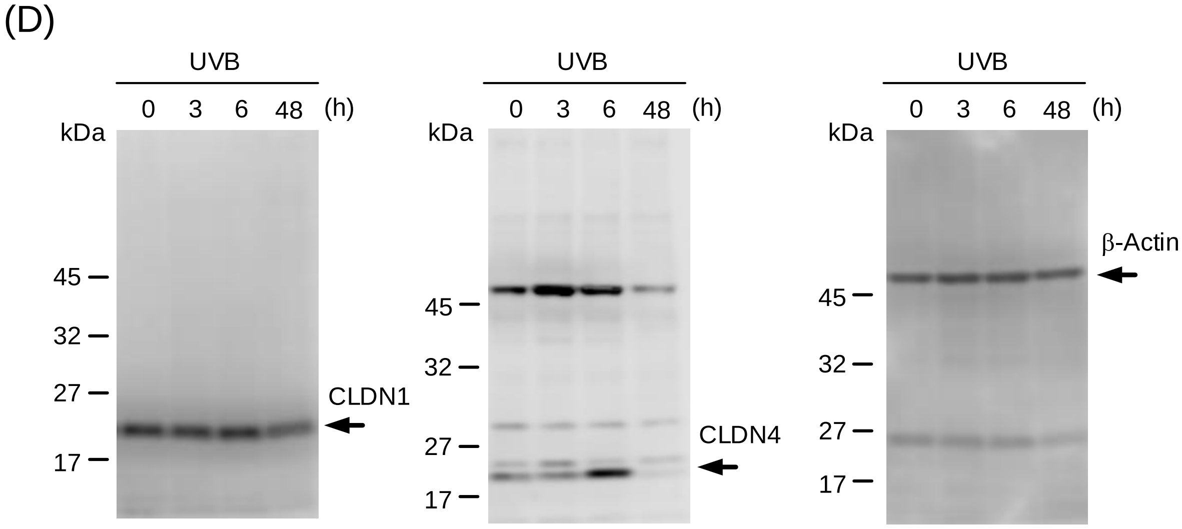

Supplementary Figure S1. Full images of the blots from figure 1.

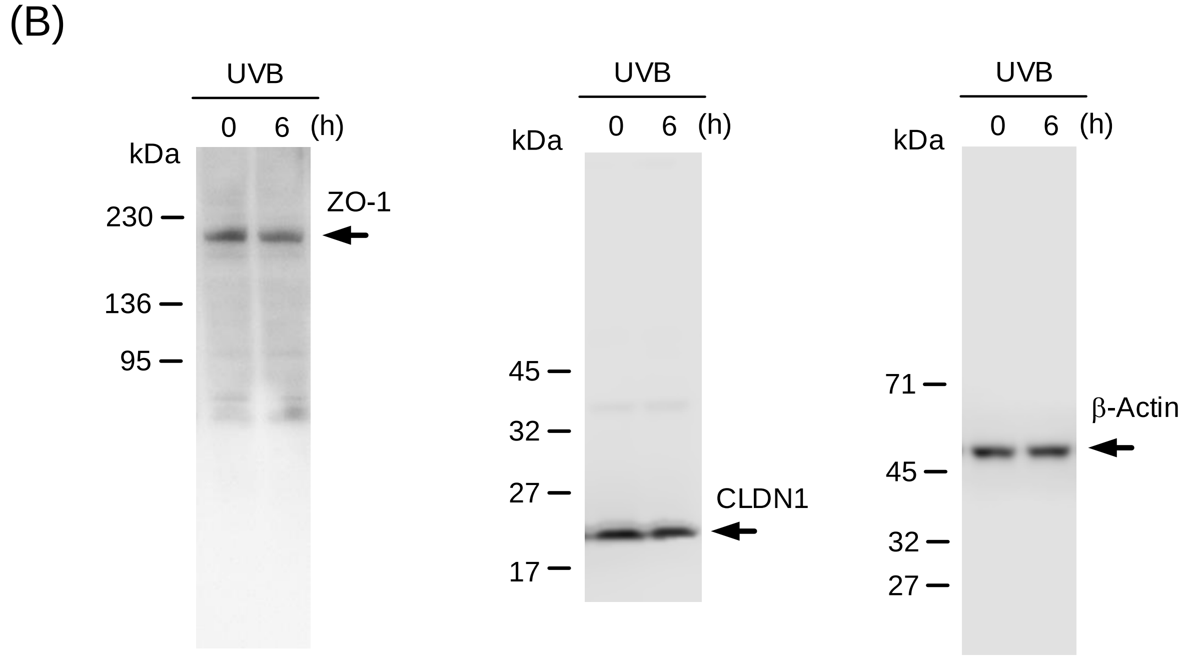

Supplementary Figure S2. Full images of the blots from figure 5B.

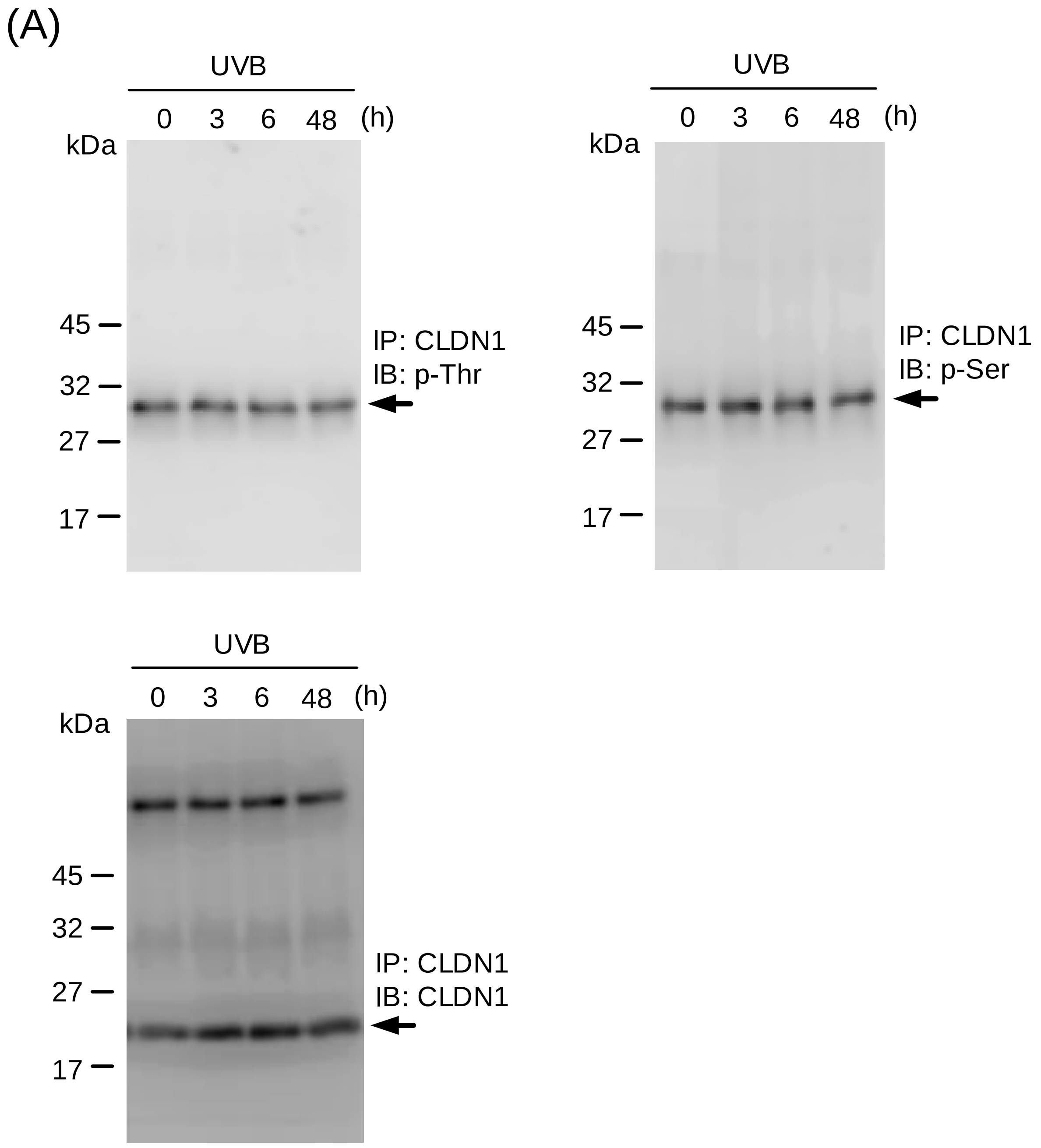

Supplementary Figure S3. Full images of the blots from figure 4A.

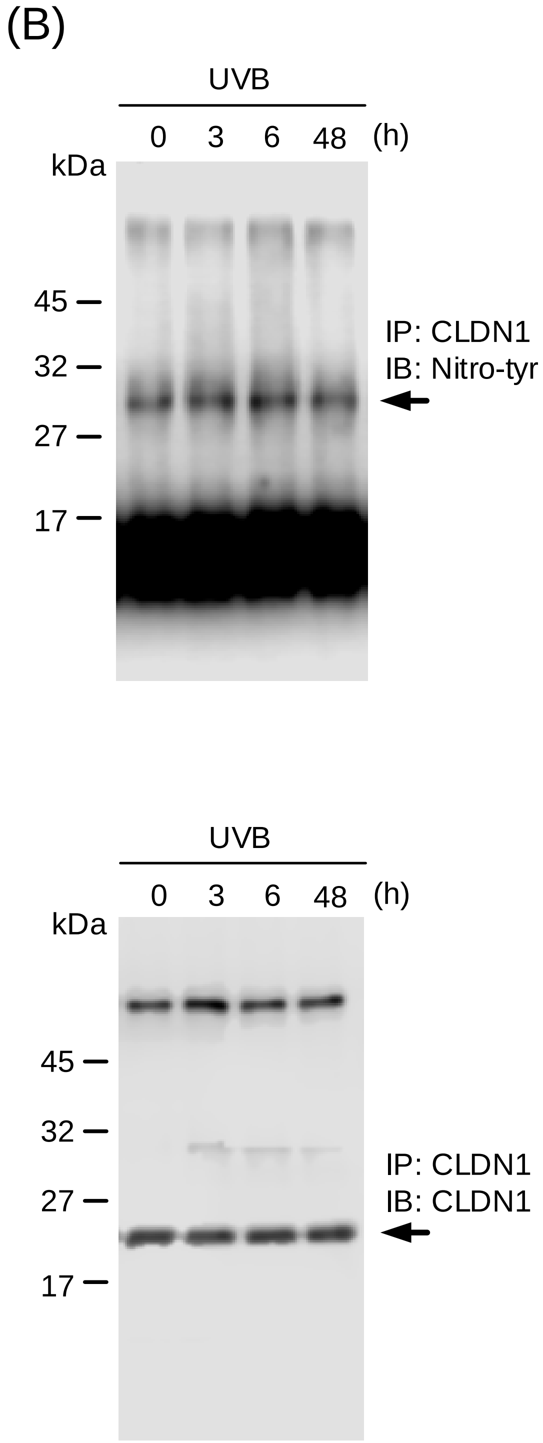

Supplementary Figure S4. Full images of the blots from figure 4B.

(C)

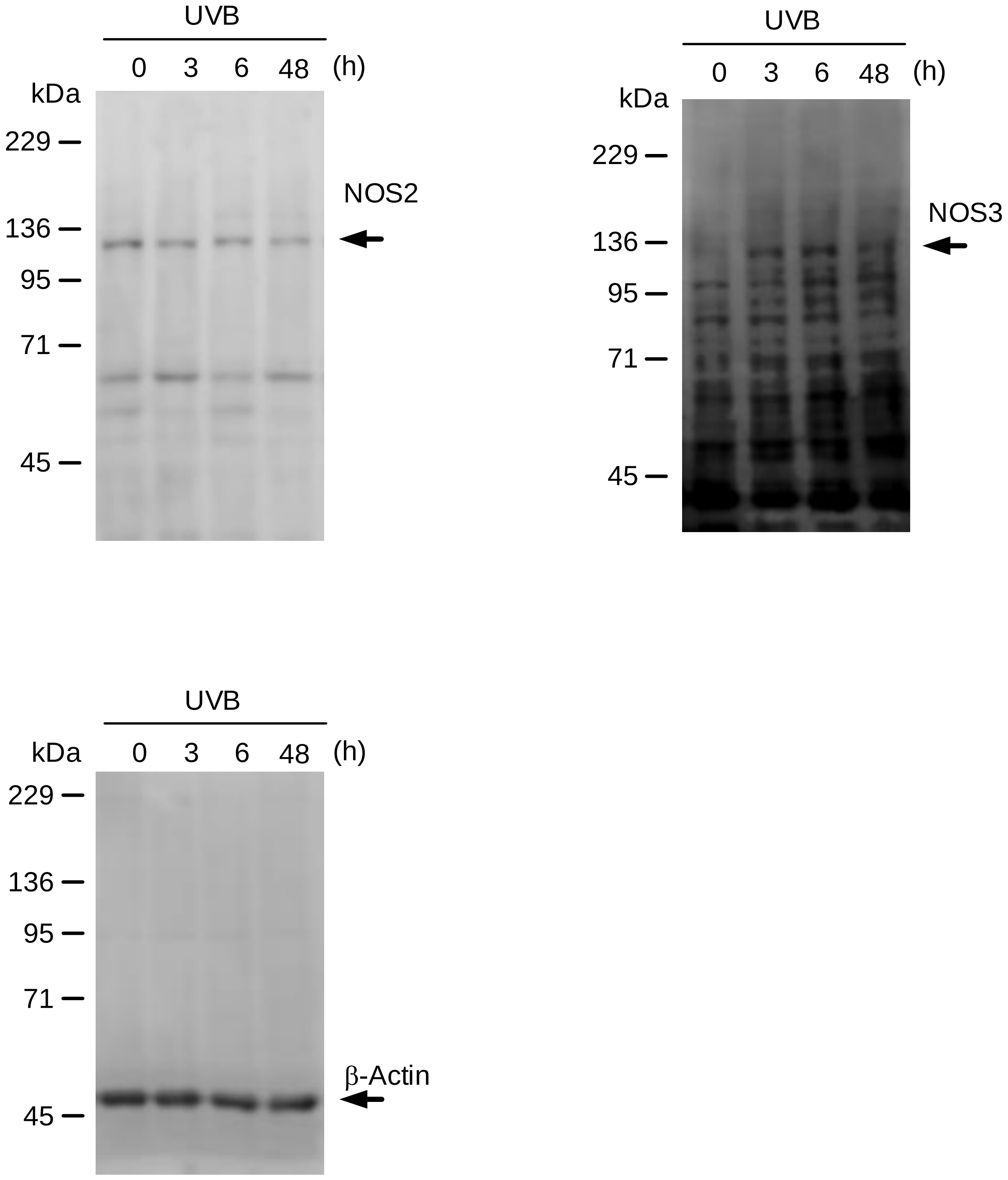

Supplementary Figure S5. Full images of the blots from figure 4C.

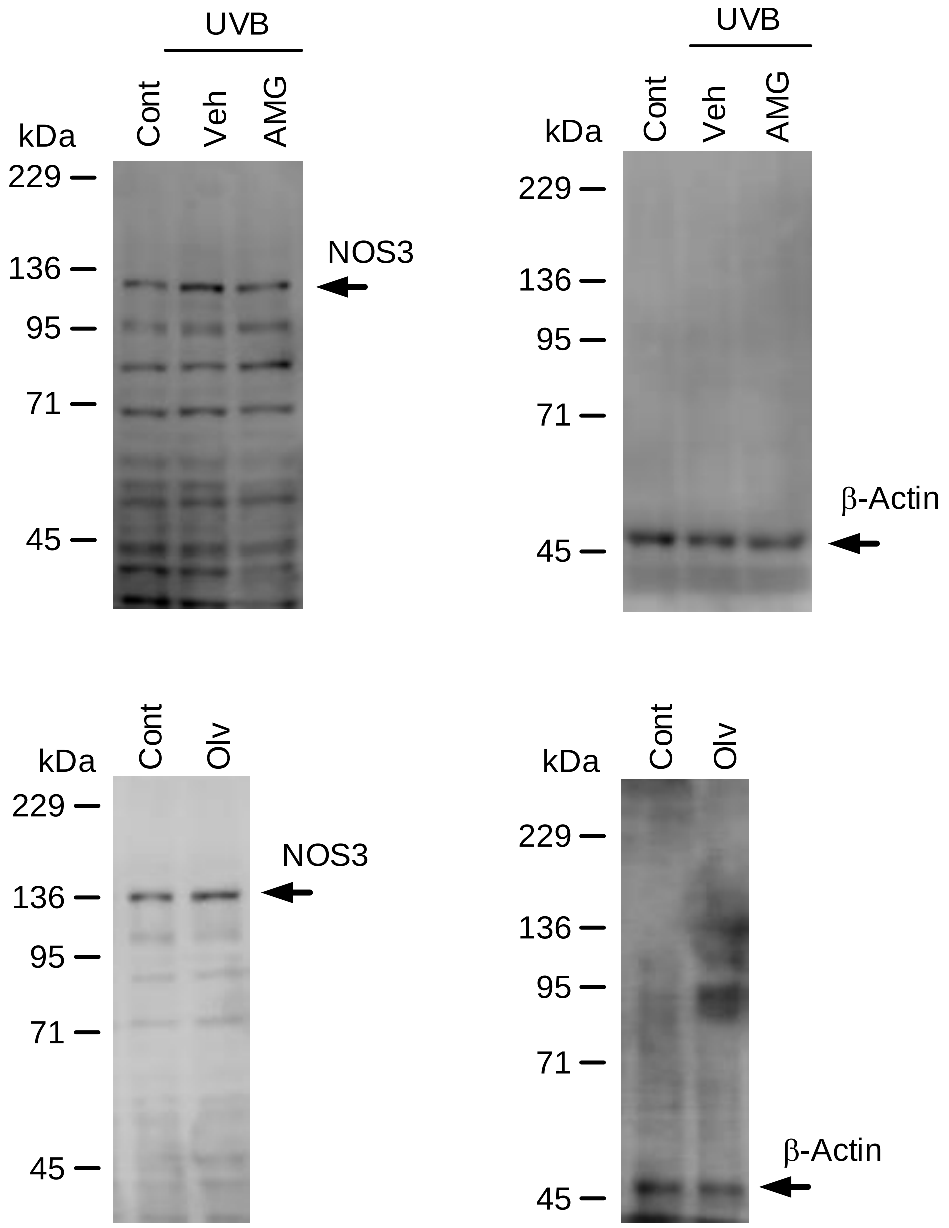

Supplementary Figure S6. Full images of the blots from figure 8.

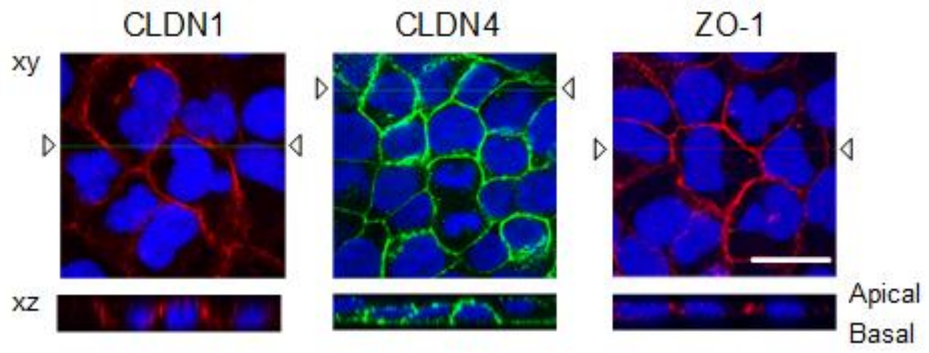

Supplementary Figure S7. HaCaT cells under control condtions were immunostained with anti-CLDN1 (red), anti-CLDN4 (green), and anti-ZO-1 (red) antibodies plus DAPI (nuclear marker). The lower panels show the vertical sections indicated by the triangles of xy images. Scale bar indicates 10 μm.

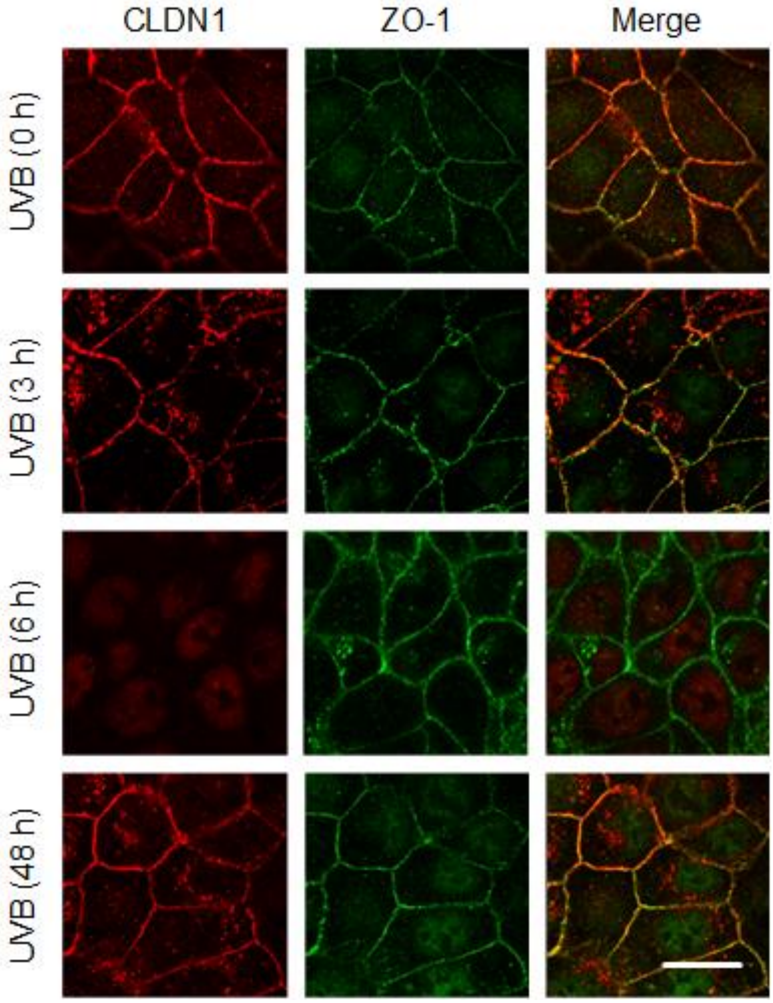

Supplementary Figure S8. HaCaT cells exposed to UVB were cultured for 0-48 h. The cells were immunostained with rabbit anti-CLDN1 (red) and mouse anti-ZO-1 (green) antibodies. The mouse anti-ZO-1 antibody was obtained from Thermo Fisher Scientific. The images were taken near the apical membrane. Scale bar indicates 10 μm.

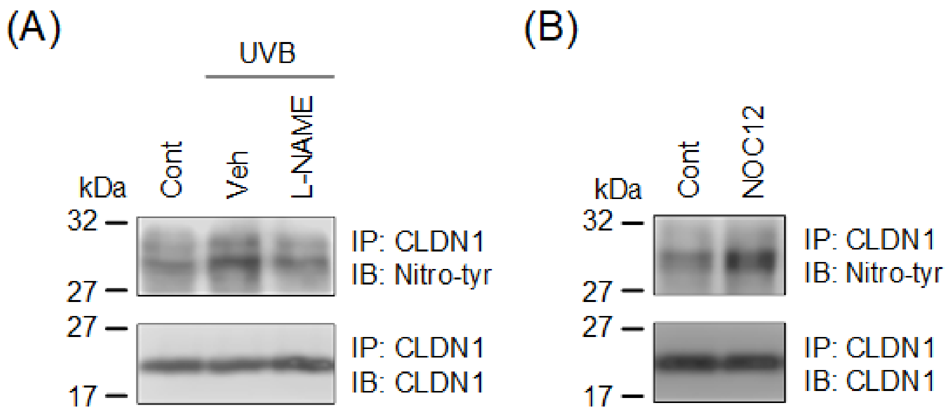

Supplementary Figure S9. (A) HaCaT cells were pre-incubated in the absence (Cont and Veh) and presence of 100 μM L-NAME for 30 min. The cells were exposed to weak UVB, followed by incubating for 6 h. (B) Cells were incubated in the absence (Cont) and presence of 10 μM NOC-12 for 6 h. After immunoprecipitation with anti-CLDN1 antibody, the immunoprecipitants were applied to SDS-PAGE, and blotted with anti-tyrosine nitration (Nitro-tyr) and anti-CLDN1 antibodies. The full-length blot images are shown in Supplementary Figure S11. n = 3.

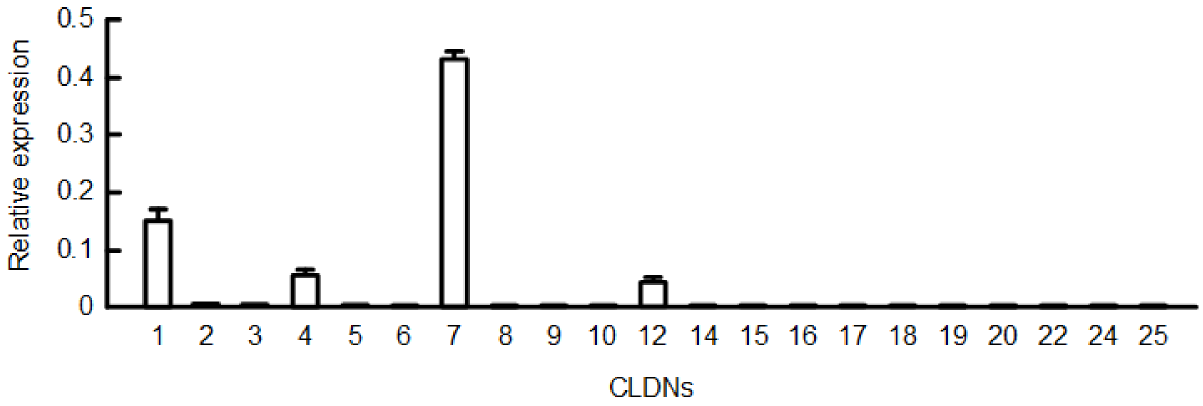

Supplementary Figure S10. Expression of mRNAs of CLDN subtypes in HaCaT cells. The expression levels of CLDN mRNAs were examined by real-time PCR. The primers used for real-time PCR are listed in Table 1 and Supplementary Table 1, and β-actin was used as the housekeeping gene. The relative expression of *CLDN* mRNA was analyzed by the ΔΔCt method. CLDN1, 4, 7, and 12 were highly expressed in HaCaT cells. The expression levels of CLDN11, 13, 21, and 23 were under detection limited.

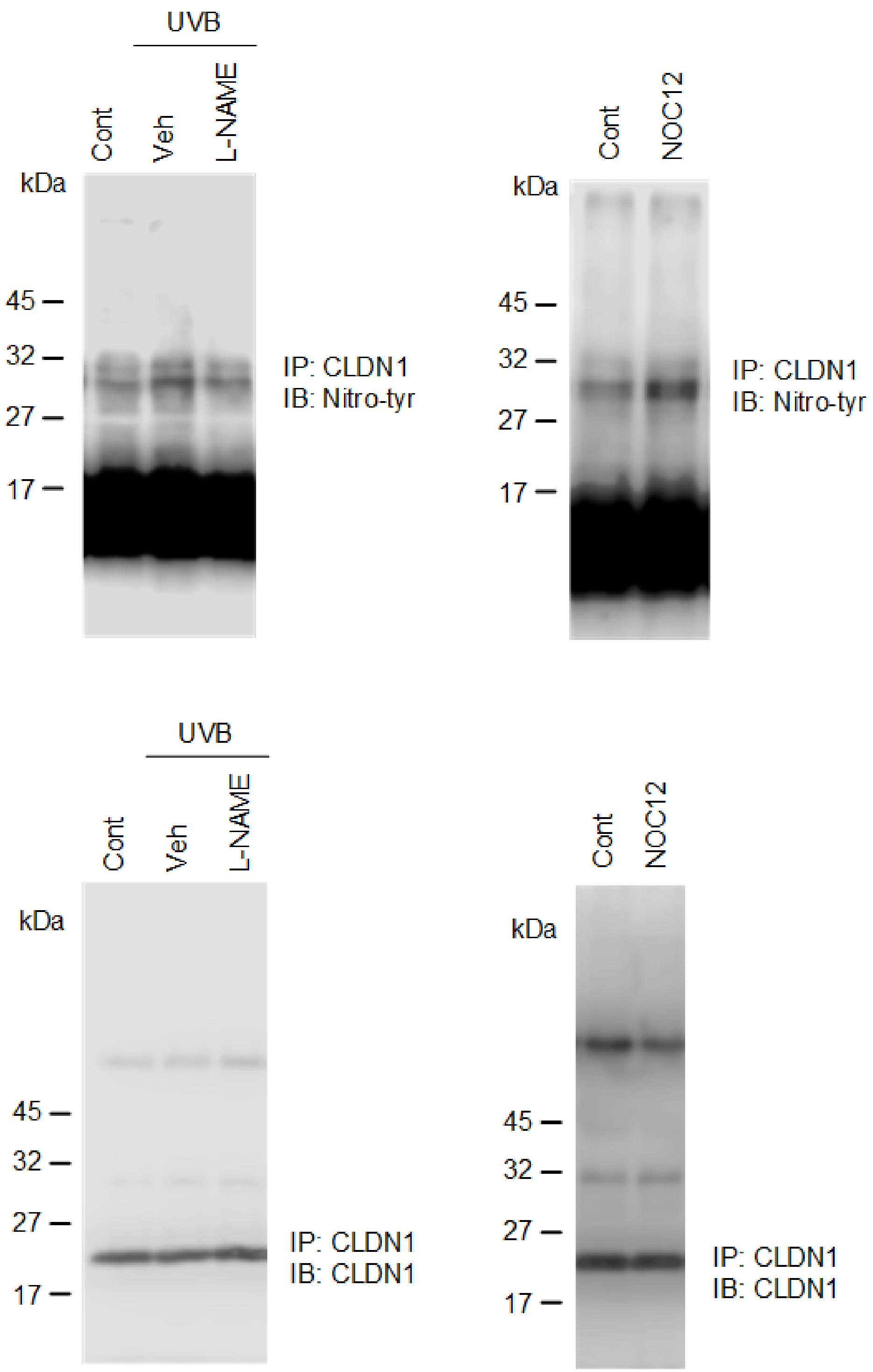

Supplementary Figure S11. Full images of the blots from Supplementary figure S8.

Supplementary Table 1. Primer pairs for real-time PCR.

Name	Direction	Sequence
CLDN2	Forward	5’-ATTGTGACAGCAGTTGGCTT-3’
	Reverse	5’-CTATAGATGTCACACTGGGTGATG-3’
CLDN3	Forward	5’-GGATGAACTGCGTGGTGCAGA-3’
	Reverse	5’-AGGATGGCCACCACGATGAG-3’
CLDN5	Forward	5’-AACATCGTGACGGCGCAGACCA-3’
	Reverse	5’-TCAGAGCCAGCACCGAGTCGTACA-3’
CLDN6	Forward	5’-GCTTTCATCGGCAACAGCATC-3’
	Reverse	5’-ACACCTTGCACTGCATCTGG-3’
CLDN7	Forward	5’-TTTTCATCGTGGCAGGTCTT-3’
	Reverse	5’-GGCCAAACTCATACTTAATGTTGG-3’
CLDN8	Forward	5’-AACTTCTGGGAAGGACTGTGGATG-3’
	Reverse	5’-GGAGAAAGAGCCAGCAGGGAA-3’
CLDN9	Forward	5’-CCTTCATCGGCAACAGCATC-3’
	Reverse	5’-CGTACACCTTGCACTGCATC-3’
CLDN10	Forward	5’-ATCGACGGCACGGTCATCAC-3’
	Reverse	5’-GGGGAAGTCCTTGCAGTTGGAG-3’
CLDN12	Forward	5’-CAACAGAAACGAGAAGAACC-3’
	Reverse	5’-ACTGATGAGTACCAAGTAGTG-3’
CLDN14	Forward	5’-ACCTGAAAGGGCTCTGGATG-3’
	Reverse	5’-AGGCAGGAGATGACCATGAG-3’
CLDN15	Forward	5’-TCCACATTCTGGCCGGTATC-3’
	Reverse	5’-CTTGGTTCCGGGGTACAAGG-3’
CLDN16	Forward	5’-CGGAGCATCCCTTGAAGCTG-3’
	Reverse	5’-GGAGCAGGGTGAGAAATCCAAAC-3’
CLDN17	Forward	5’-ATGAATTGCATCCGACAAGCCA-3’
	Reverse	5’-GCAGGGCGATCAAGGAGAGA-3’
CLDN18	Forward	5’-CCCAGGATGGTGAAATAGGG-3’
	Reverse	5’-GCAGCGAGTCGTACACCTTG-3’
CLDN19	Forward	5’-CACAGTGGAAGCAGTCTTCCTAC-3’
	Reverse	5’-GCAGCGAGTCGTAGAGCTTG-3’
CLDN20	Forward	5’-CTCCAACATCATAACAGCCATT-3’
	Reverse	5’-GACAGAATGGAGTGTTTCAGG-3’
CLDN22	Forward	5’-TGCCACACTGGAAGAACCTC-3’
	Reverse	5’-CATCCCCACTTCCTCTTGGATG-3’
CLDN24	Forward	5’-CAGGGTCTCCAGGATCTTAATG-3’
	Reverse	5’-ACAAAGTCTGGGACGTTCTCAT-3’
CLDN25	Forward	5’-AAGACTTCTGGGATGACAGCAT-3’
	Reverse	5’-AGGTCTTAGCATGAGGTGGAAG-3’
